# Ethical challenges regarding the use of stem cells: interviews with researchers from Saudi Arabia

**DOI:** 10.1186/s12910-020-00482-6

**Published:** 2020-05-12

**Authors:** Ghiath Alahmad, Sarah Aljohani, Muath Fahmi Najjar

**Affiliations:** King Abdullah International Medical Research Center (KAIMRC), King Saud Bin Abdulaziz University for Health Sciences, King Abdulaziz Medical City, Ministry of National Guard - Health Affairs, P.O. Box 22490, Mail Code 1515, Riyadh, 11426 Saudi Arabia

**Keywords:** Stem cell, Qualitative, Ethics, Informed consent, Law

## Abstract

**Background:**

With the huge number of patients who suffer from chronic and incurable diseases, medical scientists continue to search for new curative methods for patients in dire need of treatment. Interest in stem cells is growing, generating high expectations in terms of the possible benefits that could be derived from stem cell research and therapy. However, regardless of the hope of stem cells changing and improving lives, there are many ethical, religious, and political challenges and controversies that affect the research, and mandated to establish ethical guidelines and regulations. In Saudi Arabia, key stakeholders play an active role in discussing the ethics of stem cell research and therapy. The focus of the study was to explore professionals’ perceptions related to the ethical challenges of using stem cells in research and treatment in Saudi Arabia.

**Results:**

A qualitative research study was conducted to explore and describe the perceptions of 25 professionals employed at different tertiary hospitals in the various regions of Saudi. A thematic analysis was performed to search for and identify the most significant perceptions shared by the participants. Four themes were generated based on the ethical challenges of four areas related to stem cell use, including (1) forbidden and permitted sources of stem cells, (2) informed consent, (3) beneficence, and (4) ethical regulations and guidelines.

**Conclusion:**

The study identified that there is a growing need to advance the knowledge, education, and awareness related to stem cell research and treatment in Saudi Arabia.

## Background

Literature highlights the significance of understanding stem cell therapy and research to support the development of regenerative medicine [1–3]. McLaren reported that there are millions of individuals suffering from and succumbing to “incurable degenerative diseases of the nervous system, heart, liver, pancreas, and other organs” annually [3]. Similarly, Lovell-Badge discussed the fact that stem cells offer great hope for patients with enervating illnesses such as “diabetes, Parkinson’s, and Huntington’s diseases” [4]. In this context, medical practitioners consider stem cells as the hope and light for many patients who are suffering and in dire need of a cure [1]. However, as indicated by several authors, the use of stem cells, present many ethical, political, and even religious challenges, either related to resources, use, or rights of donors [5, 6]. Bharadwaj mentioned the increasing movements of social and government concerns regarding stem cell research and clinical medication in countries such as the United States, United Kingdom, and Japan. More recently, emerging countries also incorporated stem cell therapies in their practices [7].

Lo and Parham provided a classification of the different ethical issues based on the four phases of stem cell research. The first phase is the “donation of biological material,” highlighting the problem of “informed and voluntary consent” [2]. The second phase, research with human embryonic stem cells, creates several ethical issues. These issues include the “destruction of embryos and the creation of embryos for research purposes” as well as financial compensation to oocyte donors, medical hazards related to the retrieval of the oocyte, and the need to protect the reproductive interests of women undergoing infertility treatment [2]. The third phase of the research is using stem cell lines obtained from other institutions leading to the issue of adverse legal and ethical principles [2]. The fourth and last step is the use of stem cells in clinical trials, encompassing both the advantages and disadvantages of the trial and informed consent [2].

### Stem cell research in Saudi Arabia

Many Arabic countries conduct research with stem cells, as evidenced by the hundreds of scientific papers published in this field. Saudi Arabia is ahead in stem cell research as many universities, such as King Saud University and King Faisal Specialized Hospital and Research Center, started stem cell research more than 20 years ago [8]. In addition, many other research institutions, established later, play a leading role in this field such as King Abdullah International Medical Research Center with a specialized stem cell research department, including a stem cell registry containing more than 10,000 donors and the Cord Blood Bank [9].

From an ethical perspective, Saudi Arabia was the first country in the region to have ethical regulations related to the use of and research with stem cells. The Research Ethics Law promulgated in 2010 and its implementing regulation in 2012 includes all ethical guidelines to control stem cell research [10]. This was followed in 2014 by Jordan where a specific law about stem cell is announced [11]. In addition to these national laws, many research centers have institutional guidelines, for example King Faisal Specialized Hospital and Research Center and King Abdullah International Medical Research Center.

Though there is an abundance of stem cell research, the ethical component has not been researched in depth. There is no literature related to the views of physicians and researchers about the ethical challenges of using stem cells. It is important to explore this important issue to enhance the ethical component and maintaining the progress of stem cell research through finding appropriate solutions of all ethical challenges and obstacles.

## Methods

### Research design

A qualitative research design was used to explore and describe the perceptions and experiences of participants regarding the ethical challenges of using stem cells in a “subjective and reflexive manner” [12]. The aim was to gather, explore, analyze, and extract the most meaningful perceptions of the sample using interviews. The qualitative approach was deemed appropriate for the study to emphasize the content of the data to explore a specific phenomenon.

### Data collection

We collected data from professionals employed at tertiary hospitals from various regions in Saudi Arabia where stem cell research have been conducted or are being conducted. The target population included physicians from any medical specialty or non-physician researchers doing stem cell research. We visited the potential hospitals and presented the objectives and purpose of the study. We collected contact information of potential participants with the permission from the gatekeepers of the hospitals and other pertinent representatives. We used a snowball sampling technique, defined by Clark and Creswell as the sampling of individuals based on the recommendations and suggestions of others [12]. Once a participant agreed to participate, we actively inquired from the participant to identify other possible candidates for the study. The technique allowed us to recruit 25 participants. The demographic characteristics of the sample are displayed in Table 1.

**Table 1:** Characteristics of the interviewed professionals

Variables	Number
Gender of interviewees	
Male	17
Female	8
Nationality	
Saudi	17
None Saudi	8
Religion	
Islam	25
Main specialties of interviewees	
Stem cell Scientist	11
Hematologist	5
Research coordinator	5
Stem cells lab technician	3
Bioethicist	1
Type of interview	
Live	25

We conducted individual semi-structured interviews with open-ended questions and audiotaped the interviews. Before conducting the interviews, written informed consent was obtained. The consent form highlighted voluntary participation, no monetary reward or promise. The privacy and confidentiality clauses were explained thoroughly. The interviews were held in a private room at the preferred time and date of the participants. Each interview lasted between 40 and 60 min.

### Data analysis

After completing the 25 interview transcripts, data analysis commenced. The analysis involved identifying, analyzing, and reporting the most frequent and meaningful patterns or themes [12]. The analysis followed Braun and Clarke’s six-step process [13]. First, we familiarized ourselves with the interviews and actively read and reread the transcripts and generated initial codes as the second step. In the third step, we searched for themes across the data and the initial codes were categorized in the themes. We were mindful of the three objectives and purposes of the study to identify the most important points and concepts. The fourth step entailed the constant review of the themes, with the original data or the interviews. In the fifth step, the themes were named. Finally, the last step is the creation of the report as presented in the next section. We used the NVivo12 by QSR software to assist in the management and systematic tabulation of the themes.

## Results

A thematic analysis was performed to search for the most significant and meaningful responses from the sample. The thematic analysis resulted in four themes to address the three key objectives of the study. From the participant perspective, some sources are forbidden due to ethical issues. In addition, researchers and professionals must obtain an informed consent at all times (following the IRB) and follow the international regulations regarding stem cell research. The participants expressed the need to clarify the purpose of the research and storage procedures. Table 2 displays the themes in response to the study objectives.

**Table 2:** Generated study themes

**Sources of stem cell**	Permitted resources	Adult stem cells
		Pluripotent stem cells
		Umbilical cord blood
		Placenta
		Aborted fetuses for therapeutic reasons
		Spontaneously miscarried fetuses less than 120 days old
	Forbidden	Fetuses aborted for a non-therapeutic indication
		Spontaneously miscarried fetuses more than 120 days old
**Informed consent**	Importance of informed consent	
	Purpose of research should be mentioned	
	Withdrawal should be respected	
	Explanation is important and required	
	The IRB should review and approve the consent	
**Beneficence**	Any use of stem cells should be beneficial	
	Potential benefits to others	
	Should use approved procedures	
	Transparency	
	False hopes may lead to negative consequences	
**Regulations**	Following the international regulations related to stem cell research	
	Consideration of Islamic Laws (Fatwas) and Beliefs	
	Lack of the local regulation	
	Lack of adequate knowledge in researchers, and their need for training	

### Theme I: an exploration of the sample’s views regarding forbidden and permitted sources of stem cells

The participants’ position regarding the sources of stem cells can be classified in two categories: permitted and forbidden resources. The majority felt that some sources of stem cells should be forbidden because they may lead to serious religious issues, but some sources were considered safe and acceptable.

Adult stem cells as a source was considered safe providing the extraction is done within the prescribed processes and guidelines. An interviewee said, “S*ources which are like skin liver heart these are allowed… Adult stem cells- allowed.”* A second participant added, *“Adult stem cells [are] approved for clinical use.”*

Pluripotent stem cells are becoming an acceptable resource of stem cells, due to their positive and safe characteristics. An interviewee shared that the use of pluripotent stem cells is continuously advancing with the hope of curing different diseases, saying: “*Why not? It’s a new science, and the people are trying to use pluripotent stem cells in another type of... a different kind of disease, and there is a lot of clinical intervention a lot of clinical trials still under investigation there is no clear answer.”* Another participant indicated that pluripotent stem cells are similar to adult stem cells and can easily be replicated, saying: *“Pluripotent stem cells it’s actually an adult cell you reprogramed the genetic and you move it back so you can do anything with it.”*

According to our interviewees, the umbilical cord is a safe and promising stem cell source. A participant commented, *“umbilical cord is one of the best sources of stem cells.”*

The participants also indicated that the placenta is a permitted source, and they experienced no issues as the placenta was used previously to extract stem cells. A participant stated that using the placenta is not harmful, saying, *“We used to collect stem cells from the placenta. It is not invasive.”*

Obtaining stem cells from fetuses were perceived differently. One group clearly and completely forbids any use of stem cells from any fetus, either intentionally aborted or accidently miscarried, regardless of the age of fetus. One of the interviewees responded that the use is strictly prohibited, stating, *“This is forbidden”* and a second indicated clearly and strongly, *“Miscarried fetuses before reaching [120 days] the same, the same, forbidden.”* This group of professionals stated that the aborted fetus is rejected as the institutions and stakeholders are aware that the use of such a source may lead to ethical dilemmas. As one of the participants expressed, *“there may be ethical issues that go along with the use of an aborted fetus.”* In addition, the use of embryonic stem cells may lead to more serious and critical religious issues. A participant stated, *“That they must adhere to the religious teaching that one must not touch or alter the fetus.”*

However, some of the participant accepted fetuses aborted for therapeutic reasons, but forbid stem cells obtained from fetuses aborted for non-therapeutic reasons. An interviewee said, *“However, these source of stem cells that we’re using is probably less chaotic, and hence should be utilized or the regulations should be applied like any other biological materials.”* Spontaneously miscarried fetuses can be accepted as a resource of stem cells, if they are less than 120 days of age. One interviewee said, *“If the fetus is less than 120 days old, it is not considered a human, and we can use its stem cells.”*

### Theme II: an exploration of professionals’ opinions regarding the ethical challenges of securing informed consent, with IRB approval

The second thematic category explored the sample’s perceptions concerning the challenges related to obtaining informed consent for stem cell research. The participants emphasized the importance of informed consent to guarantee the voluntary participation of donors. One of the interviewees said, *“We do have consent actually, we never collect stem cells without taking a consent from the patient. Sure, sure yeah, we take the permission before we start collecting the cells.”* However, for umbilical cord blood, consent is obtained from parents, usually during the routine visits to clinics during the pregnancy, as expressed by one interviewee.

According to the participants, the consent should explain and clearly describe the purpose of the research. A participant said: *“I think that the donor should be informed what exactly we are doing with tissue that he has that we take it from him.” Another said, “The scope of the research should be properly presented. Such practice will protect both parties, the researcher and donor, from future issues.”*

All donor rights should be mentioned in the informed consent. One of the interviewees said, *“Donors’ rights musts be clarified and explained to them, including, but not limited to, withdrawal right.”*

The explanation should be in understandable, clear language and the terms and conditions of the forms should be simplified. The communication must be sufficient to ensure the donor understand fully. A participant narrated, *“The researchers must take the time to orient and explain the content of the informed consent to the volunteers.”* The informed consent documents should have been reviewed and approved by an ethics committee. A participant discussed the process of procuring the form, as follows: *We submit the consent to the research office as a part of the submitted research proposal and then you will get IRB approval for all proposals including the informed consent.”*

### Theme III: an exploration of the professionals’ perceptions regarding the ethical challenges related to the benefit resulting from the stem cell research

The participants described the potential value of the sources of stem cells in the field of medicine and research. According to the majority, any use of stem cells should be beneficial, either to the donors or to the public. A participant said, *“Even if there is no direct benefit to the donors themselves, but at least stem cells research should have some potential benefits to others.”* A second opinion was *“There are different applications and uses of the umbilical cord and it can save many patients today.”* When using stem cells in treatment, the approved procedures should be followed meticulously, as explained by one of the interviewees, *“Not following approved methods may lead to serious consequences.”*

The sample emphasized the responsibility of being transparent when informing and communicating with the patient about any potential benefit. *“It is very important not give the patients false hope about treatment by stem cells,”* as expressed by one of the interviewees. A second participant was concerned about false hopes based on wrong assumptions, *“I am very sad to see hopeless people spend all their earnings and energy in trying something that can never be a success.”*

### Theme IV: an exploration of stem cell research regulations

Four subthemes were developed related to the regulations related to stem cell research theme, including the importance of following international regulations, the need to use international guidelines based on Islamic laws (fatwas) and beliefs, no national law related to stem cells, and the need to increase the researchers’ knowledge about the ethical guidelines of stem cells.

The majority of the sample considered following regulations and guidelines consistently every time stem cells are used as an important issue. A participant explained, “*We have to follow the set procedures by regulators and the law because it involves safety of patients.”* Our findings indicate that our researchers are using international regulations in their current practice and research with stem cells. One researcher said, “*Actually we are already use international guidelines. We used to follow that when I was getting my training in the west, and we here continue do the same.”* Another researcher justified why international regulations should be followed, *“Following the international regulations on stem cell research is needed for two reasons: the ethical principles are the same, and we are in many cases part of international multicenter research.”* However, when applying these international regulations, Islamic law and fatwas should be taken in account. One of the participants explained, *“Nothing that contradicts Sariah is acceptable, and this is true when it comes to stem cells, especially when it comes to the permitted or forbidden resources.”*

The participants also indicated a lack of standardization in the local setting. A participant narrated that currently, they follow only the international regulations for their clinical trials and research. The participant described the increasing need for a targeted local policy related to stem cells, saying: *“There is no national standardization as far as I know.”* According to a second participant, the main issue in Saudi is the actual lack of a national law related to stem cell research and therapy, he commented, *“Ethics must be a priority in Saudi to be able to create laws that would be in line with the local religious or spiritual beliefs.”* Another participant also expressed the need to create local guidelines, which should match the international guidelines and not contradict the Islamic law. A last perspective was as follows: *“Definitely, it’s good to have a supporting in fatwa for our patients satisfaction. This is because patients and the community rely on the fatwa more than the IRB. They don’t know about the IRB. So, I think that’s why the need the fatwa. I think we would reassure our patients about that.”*

Many participants admitted that researchers lack adequate knowledge and information regarding ethical considerations and guidelines about stem cells research. One of the participants said, *“The researchers themselves need to be trained or oriented in a formal setting to become aware about the ethical guidelines of using stem cells.*”

## Discussion

Though researchers are doing research in the stem cell field, they realize the ethical challenges they are facing in their research. Having spent a significant part of their scientific life in Western countries, they are aware of ethical issues; however at the same time, their cultural and religious background plays a role in their perceptions regarding the ethical challenges and how to deal with them.

The first point to manage appropriately is the source of stem cells, classified in permitted and forbidden sources. While the sample accepts adult stem cells in general, they have a different point of view regarding embryonic stem cells. These stem cells are affected by ethical, legal, and religious considerations, especially regarding the method of obtaining the cells and more specifically, when it results in destroying embryos who may have a degree of dignity and humanity, similar to other researchers, societies and universities in the west [14]. A particular concern is if the fetus is more than 120 days old, the time of soul installment according to Islamic law [15], which is in the middle between the two opposing opinions: the first sees embryos less than complete and conscious persons, while the second sees them equal to all human beings and should not be treated differently. The sensitivity related to using stem cells from embryonic sources resulted in a significant increase in the interest of adult stem cells in medical research, even though the lesser importance they have.

The researchers’ points of view about permitted and forbidden sources, as stated in the Saudi law of ethics of research on living creatures and its implementing regulations [10], match almost completely except for limiting the use of pluripotent stem cells to laboratories only. The researchers did not mention extra fetuses (extra fertilized eggs) which, as prescribed in the Saudi law, are not permitted as a source of stem cells. The reason may be because it is neither a common practice nor legal to use this source. Researchers where satisfied with the available sources, namely cord blood and imported cell lines.

The source of stem cells was not the only point discussed by the sample. They highlighted several factors to be considered to ensure stem cell research is ethical. The points are a component of the general rules related to conducting ethical medical research, locally and internationally [16]. It was expected that the sample would mention obtaining informed consent prior to any stem cell donation, adult or embryonic, before use. Informed consent should also be obtained from donors of adult stem cells. However, for embryonic stem cells, consent should be obtained from the parents. It is noteworthy that the researchers highlighted the importance of clarifying the purpose of stem cell donation to the potential donors to avoid any possibility of employing practices that may invalidate the consent. Mandating review and approval of an ethics committee of the informed consent form protect the donors who may miss understanding some points in the informed consent documents. Although there is no direct benefit to the donors from stem cell research, the altruism principle is an important motivation to donate stem cells, which is supported by studies in other regions [17]. The participants mentioned the importance that research should not be futile but have a direct or indirect benefit. The researchers recognize that stem cells have the potential of future success and many people, especially patients with chronic diseases and difficult to treat diseases, have placed their hope on stem cells. So, it is understandable that the sample mentioned repeatedly that patients should be warned against false hope and they emphasized the importance of transparency in stem cell treatment or research; the idea that is highlighted by other researchers [18].

The awareness of researchers about the importance of respecting and complying to international guidelines can be understood in the context of receiving tertiary education abroad where they internalized the international guidelines and conducted research according to these guidelines. Frequently, the current research in Saudi Arabia is a continuation of their previous research during their training. The second reason which explains the importance of following international guidelines is that the majority of research is multi-center international studies and following the same principles is essential for success.

The harmonization of ethical and legal rules related to stem cell research with the Islamic point of view is important due to two reasons. Firstly, the acceptance or willing participation of potential donors will significantly increase if they are informed that the research is in line with Islamic law, and secondly, the Research Ethics Law in Saudi Arabia mandates that all practices in stem cell research should be in line with Islamic rules to be allowed and legitimate. However, the sample where not sufficiently aware of the regulation related to stem cell research mentioned in the Saudi Law of Research Ethics [10]. This caveat reflects a lack of responsibility about keeping themselves updated, as the law is readily available on the website of the National Committee of Bioethics www.kacst.edu.sa. From another perspective, the offices responsible in the National Committee of Bioethics should promote the law efficiently to raise awareness in researchers and donors.

## Conclusion

In conclusion, the participants of the study indicate various ethical challenges regarding the use of stem cells in research. For the majority of the participants, specific stem cell sources are forbidden in Saudi. Particularly, embryonic stem cells as the use may result in serious religious issues. The participants also reject aborted or (some) miscarried fetuses as a source. In response to the second objective, the ethical principles and challenges related to stem cell research were identified. The sample emphasized the importance of always securing IRB approval of the informed consent documents. Informed consent should include an explanation of the scope of the research and the participants’ rights, in simple understandable language to ensure complete understanding.

The majority of the participants reported that they already follow the international regulations related to stem cell research, which they had been exposed to during their studies and training, mostly in Western countries. However, surprisingly, they are not necessarily aware of existing national local laws, which reflects a critical need of research ethics education in general and in stem cell ethics in particular, through courses, conferences, and university programs-which are currently lacking in Saudi. Also, conducting analytic and comparative studies about stem cells in Saudi research ethics law may help to increase awareness among researchers. Additional in-depth research to include different categories with different levels will be very important at the next stage.

## Data Availability

The datasets generated during the study are available from the corresponding author on reasonable request.
